# Happiness

**Published:** 1874-03

**Authors:** 


### HAPPINESS.

A PEASANT BOY once said he would be perfectly happy, if he had nothing to do all day but to swing on the gate and eat molasses.

The poet Gray is reported to have declared, that his highest conception of enjoyment, was to lie all day on a sofa and read romances.

Dr. Scudder, the great and good missionary, tells of one of his heathen pupils of seven years, that she said to her mother one day:

"Mother, I have found out how to be happy."

"How, my dear child?"

"By trying to do all I can to make others happy."

When a child of a dozen years we succeeded, after a long trial, in making and placing a marten-box on a building near our honored father's dwelling. The twitterings of this beautiful bird of a summer's morning, add no little life to the quiet of a country village. As vivid, as if it were but yesterday, is the recollection of the feeling that we would be perfectly happy if the martens would only come to our box. Happy for us, if our after ambitions had been as innocent as that of our childhood's summer.

